# Gene therapy for the leukodystrophies: From preclinical animal studies to clinical trials

**DOI:** 10.1016/j.neurot.2024.e00443

**Published:** 2024-09-13

**Authors:** Jasna Metovic, Yedda Li, Yi Gong, Florian Eichler

**Affiliations:** a Department of Neurology, Massachusetts General Hospital, Harvard Medical School, Boston, MA 02114, USA; b Center for Genomic Medicine, Massachusetts General Hospital, Boston, MA, USA

**Keywords:** Leukodystrophies, Gene therapy, Adeno-associated viral vector, Lentiviral vector, Antisense oligonucleotide

## Abstract

Leukodystrophies are progressive single gene disorders affecting the white matter of the brain. Several gene therapy trials are in progress to address the urgent unmet need for this patient population. We performed a comprehensive literature review of all gene therapy clinical trials listed in www.clinicaltrials.gov through August 2024, and the relevant preclinical studies that enabled clinical translation. Of the approximately 50 leukodystrophies described to date, only eight have existing gene therapy clinical trials: metachromatic leukodystrophy, X-linked adrenoleukodystrophy, globoid cell leukodystrophy, Canavan disease, giant axonal neuropathy, GM2 gangliosidoses, Alexander disease and Pelizaeus-Merzbacher disease. What led to the emergence of gene therapy trials for these specific disorders? What preclinical data or disease context was enabling? For each of these eight disorders, we first describe its pathophysiology and clinical presentation. We discuss the impact of gene therapy delivery route, targeted cell type, delivery modality, dosage, and timing on therapeutic efficacy. We note that use of allogeneic hematopoietic stem cell transplantation in some leukodystrophies allowed for an accelerated path to clinic even in the absence of available animal models. In other leukodystrophies, small and large animal model studies enabled clinical translation of experimental gene therapies. Human clinical trials for the leukodystrophies include *ex vivo* lentiviral gene delivery, *in vivo* AAV-mediated gene delivery, and intrathecal antisense oligonucleotide approaches. We outline adverse events associated with each modality focusing specifically on genotoxicity and immunotoxicity. We review monitoring and management of events related to insertional mutagenesis and immune responses. The data presented in this review show that gene therapy, while promising, requires systematic monitoring to account for the precarious disease biology and the adverse events associated with new technology.

## Introduction

Leukodystrophies are heritable, progressive disorders that predominantly affect the white matter of the central nervous system (CNS). As a group, the overall incidence is one in approximately 7600 live births [1]. All leukodystrophies affect myelin, the insulation around nerves that enables rapid communication between neurons. Pathologic processes destroy existing myelin (demyelination), trigger abnormal myelin deposition (dysmyelination), or prevent myelin deposition (hypomyelination) in the CNS and/or peripheral nervous system (PNS) during development [2]. Currently, more than 50 disorders are classified as leukodystrophies, and this number continues to grow. Mortality is 34% with an average age at death of 8.2 years [1]. While clinical manifestations can be highly variable even for patients with the same leukodystrophy, disease severity is often inversely correlated with age at disease onset. The most severe forms present in infancy with rapid progression to neurologic devastation and death.

Treatment approaches vary based on pathogenesis, but most fall into one of four categories: 1) enzyme replacement therapies replace the missing or defective enzyme; 2) substrate reduction therapies reduce the buildup of the (often toxic) compound that cannot be adequately metabolized; 3) cell therapies replace diseased cells with healthy allogeneic cells or corrected autologous cells; 4) gene therapies functionally replace the missing or defective gene. In this review, we focus on gene therapies that have reached clinical trials as we aim to understand studies needed to enable leukodystrophy trial readiness. Prior leukodystrophy reviews focus on cellular mechanisms [3] and available clinical trials [4]. Here, we present an updated and comprehensive review of all gene therapy clinical trials for leukodystrophy patients in the context of disease-specific pathophysiology and preclinical studies.

### Classification and cellular mechanisms underlying the pathogenesis of leukodystrophies

**Fig. 1:**
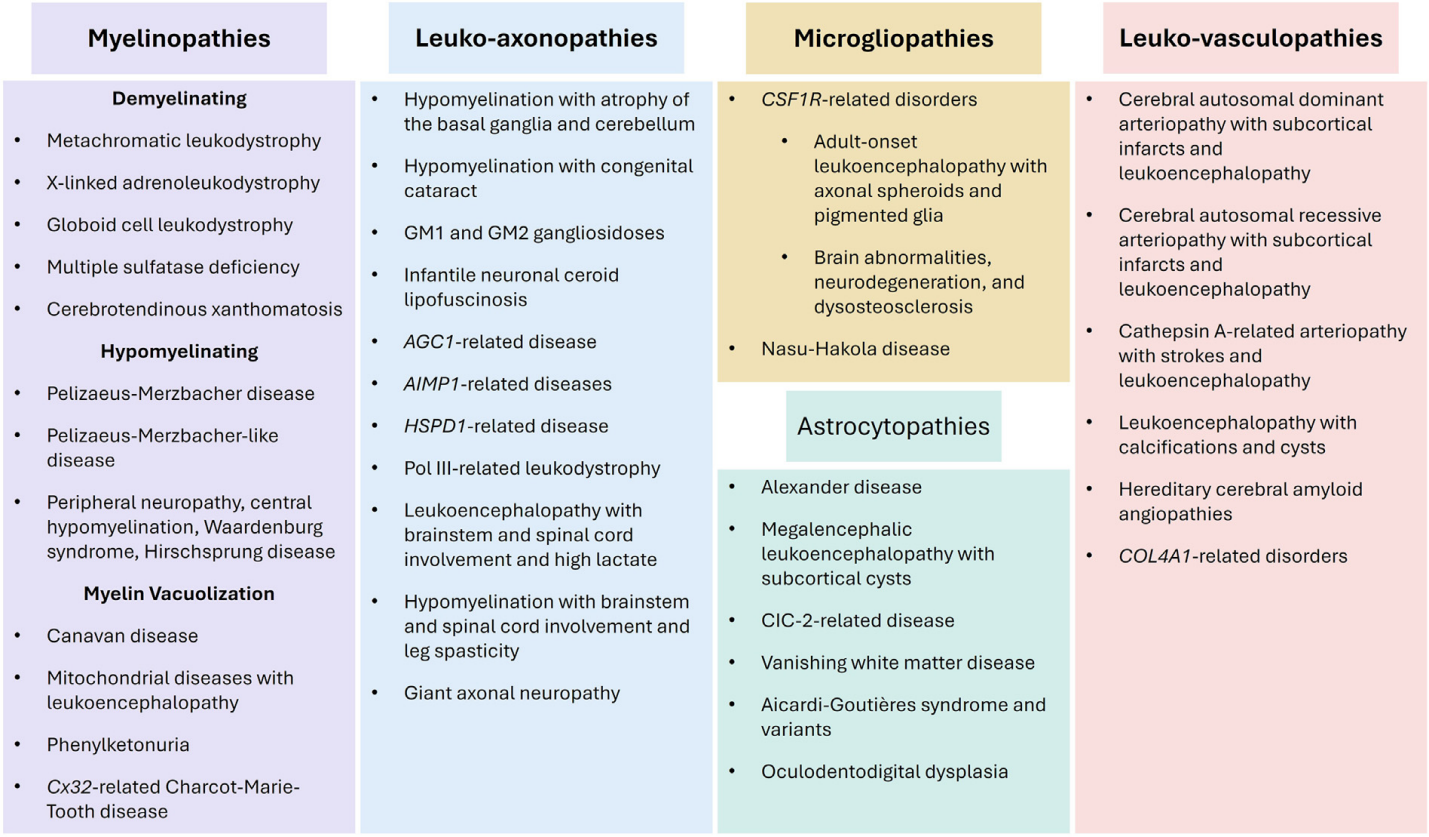
A classification system for leukodystrophies based on cellular mechanisms and pathophysiology, adapted from van der Knaap and Bugiani [2]. This is not a comprehensive list of the leukodystrophies.

The definition of leukodystrophies has evolved significantly with advances in next generation sequencing, magnetic resonance imaging (MRI), and molecular biology techniques that together have informed a more nuanced understanding of pathophysiology [3,5,6]. In 2017, van der Knaap and Bugiani [2] proposed a new classification system that grouped leukodystrophies into five categories related to pathologic changes and pathogenic mechanisms (Fig. 1, Table 1). Myelinopathies arise from defects in oligodendrocytes or myelin structure. They are further subcategorized as disorders of hypomyelination, demyelination, and myelin vacuolization which disrupts myelin integrity. Leuko-axonopathies stem from defects in neurons and their axonal processes. Astrocytopathies and microgliopathies disrupt neuroinflammation and CNS repair. Leuko-vasculopathies develop from pathologic processes within the small blood vessels of the brain [2]. Some pathologically relevant CNS cell types, such as microglia, can derive from hematopoietic stem cell precursors with important implications for therapeutic development.

**Table 1:** List of common leukodystrophies, their associated genes, inheritance, and pathophysiology.

Disease	Gene affected/cell type	Inheritance	Gain or Loss of Function	Pathophysiology	Predominant cell type(s) affected
**MYELIN DISORDERS**					
**Demyelination**					
X-linked adrenoleukodystrophy	*ABCD1*	XLR	LOF	Impaired transport of very long chain fatty acids into the peroxisome	Oligodendrocytes, neurons
Metachromatic leukodystrophy	*ARSA*	AR	LOF	Impaired lysosomal sulfatide metabolism	Oligodendrocytes, microglia
Globoid cell leukodystrophy	*GALC*	AR	LOF	Impaired metabolism of galactosylsphingosine in the lysosome	Oligodendrocytes
Cerebrotendinous xanthomatosis	*CYP27A1*	AR	LOF	Impaired cholesterol metabolism	Oligodendrocytes
**Hypomyelination**					
Pelizaeus Merzbacher disease	*PLP1*	XLD	GOF	Accumulation of mutant PLP1 in the endoplasmic reticulum	Oligodendrocytes
Pelizaeus Merzbacher like-disease	*GJC2*	AR	LOF	Impaired gap junction formation and cell-cell communication	Oligodendrocytes, astrocytes
**Myelin vacuolization**					
Canavan disease	*ASPA*	AR	LOF	Impaired lysosomal metabolism of N-acetylaspartic acid	Oligodendrocytes
*Cx32*-related Charcot-Marie-Tooth disease	*GJB1*	XLR	LOF	Impaired stabilization of myelin structure, impaired calcium signaling	Oligodendrocytes
**ASTROCYTOPATHIES**					
Alexander disease	*GFAP*	AD	GOF	Toxic accumulation of mutant GFAP, impaired intermediate filament formation	Astrocytes
Megalencephalic leukoencephalopathy with subcortical cysts	*MLC1* *HEPACAM*	AR AR/AD	LOF	Abnormalities of cell junctions, impaired astrocyte-astrocyte communication	Astrocytes
Vanishing white matter disease	*EIF2B1; EIF2B2; EIF2B3; EIF2B4; EIF2B5*	AR	LOF	Abnormal messenger RNA translation, impaired cellular stress response	Astrocytes, oligodendrocytes
Aicardi-Goutières syndrome	*ADAR* *RNASEH2A* *RNASEH2B* *RNASEH2C* *SAMHD1* *ATREX1* *IFIH1*	AR/AD AR AR AR AR AR/AD AD	LOF LOF LOF LOF LOF LOF LOF	Abnormal nucleic acid metabolism OR abnormal immune system activation	Astrocytes, oligodendrocytes
Oculodentodigital dysplasia	*GJA1*	AD/AR	LOF	Abnormal connexin 43 structure, irreversible gap junction closure	Astrocytes, oligodendrocytes
**LEUKOAXONOPATHIES**					
*TUBB4A-*related leukodystrophy	*TUBB4A*	AD	GOF	Impaired formation and stability of microtubules	Neurons, oligodendrocytes
Hypomyelination with congenital cataract	*FAM126A*	AR	LOF	Abnormal myelin synthesis	Neurons
Gangliosidosis GM1	*GLB1*	AR	LOF	Impaired lysosomal metabolism of gangliosides	Neurons, oligodendrocytes
Gangliosidosis GM2	*HEXA* *HEXB*	AR AR	LOF	Impaired lysosomal metabolism of gangliosides	Neurons
Pol-III related disorders	*POLR3A* *POLR3B*	AR AR	LOF	Impaired assembly and function of RNA polymerase III	Neurons, oligodendrocytes
Hypomyelination with brainstem and spinal cord involvement and leg spasticity	*DARS1*	AR	LOF	Impaired aspartyl-tRNA synthetase function	Neurons
Leukoencephalopathy with brainstem and spinal cord involvement and lactate elevation	*DARS2*	AR	LOF	Impaired aspartyl-tRNA synthetase function in mitochondria	Neurons, astrocytes
Giant axonal neuropathy	*GAN*	AR	LOF	Impaired ubiquitin-proteosome function, impaired neurofilament degradation	Neurons
**MICROGLIOPATHIES**					
*CSF1R-*related leukoencephalopathy	*CSF1R*	AD AR	LOF (HIS) LOF	Impaired auto-phosphorylation of CSF1R tyrosine kinase and microglial function	Microglia
Polycystic lipomembranous osteodysplasia with sclerosing leukoencephalopathy (Nasu-Hakola disease)	*TYROBP* *TREM2*	AR AR	LOF LOF	Abnormal microglial and osteoclast activation	Microglia
**VASCULOPATHIES**					
Cerebral autosomal dominant arteriopathy with subcortical infarcts and leukoencephalopathy	*NOTCH3*	AD	GOF	Impaired function and survival of vascular smooth muscle cells	Vascular smooth muscle cells
Cerebral autosomal recessive arteriopathy with subcortical infarcts and leukoencephalopathy	*HTRA1*	AR	LOF	Abnormal TGFβ signaling, abnormal angiogenesis	Vascular smooth muscle cells

Abbreviations: AD: autosomal dominant; AR: autosomal recessive; GOF: gain of function; HIS: haploinsufficiency; LOF: loss of function; XLD: X-linked dominant; XLR: X-linked recessive.

**Fig. 2:**
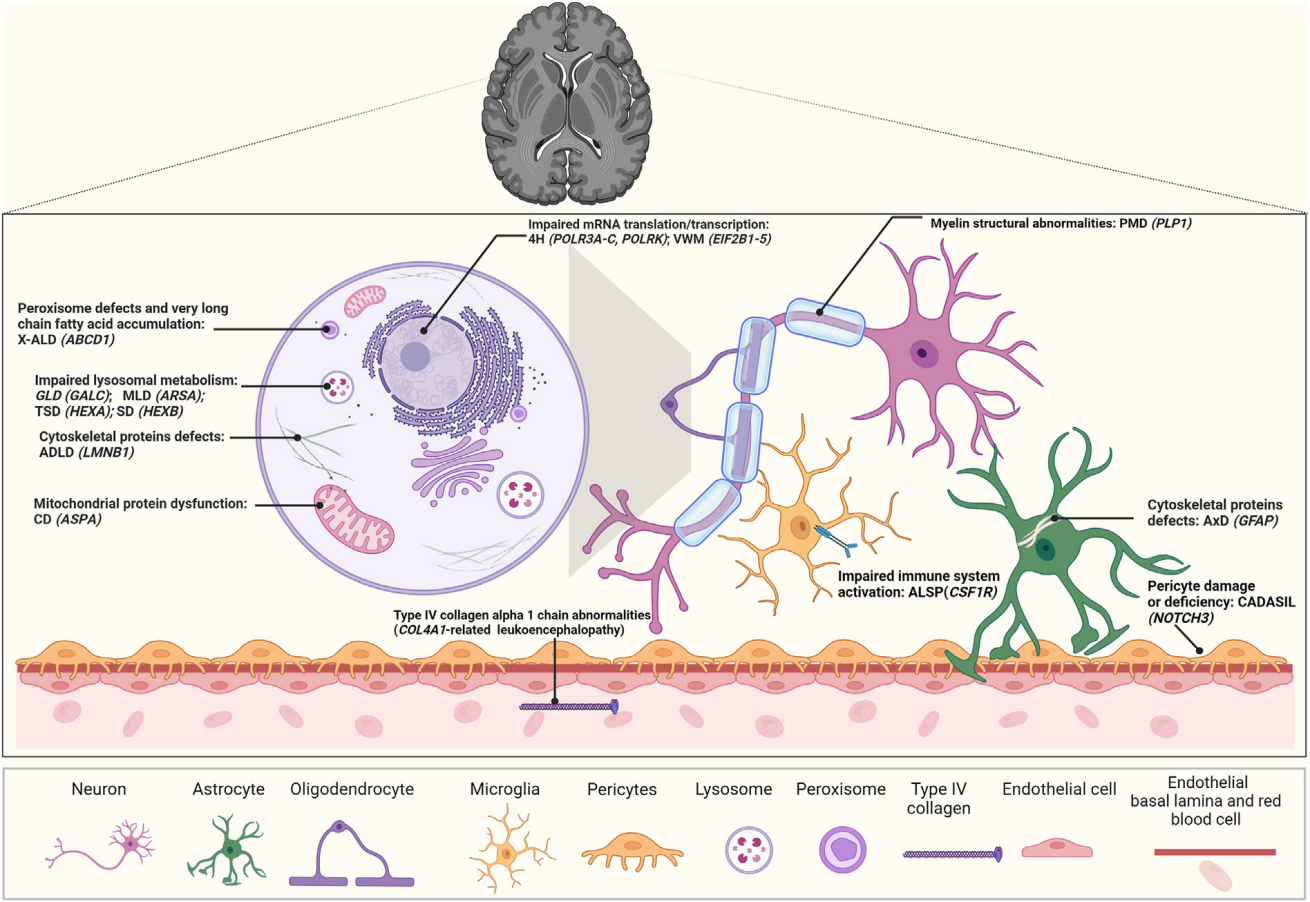
Molecular and cellular mechanisms disrupted by leukodystrophy pathophysiology in the setting of the brain tissue microenvironment. Abbreviations: 4H: Hypomyelination with hypogonadotropic hypogonadism and hypodontia or *POL-III* related disorders; X-ALD: X-linked adrenoleukodystrophy; GLD: Globoid cell leukodystrophy; MLD: Metachromatic leukodystrophy; TSD: Tay-Sachs disease; SD: Sandhoff disease; ADLD: Autosomal dominant leukodystrophy; CD: Canavan disease; ALSP: Adult-onset leukoencephalopathy with axonal spheroids and pigmented glia (*CSF1R*-related leukoencephalopathy); PMD: Pelizaeus-Merzbacher disease; AxD: Alexander disease; CADASIL: Cerebral autosomal dominant arteriopathy with subcortical infarcts and leukoencephalopathy.

Pathogenic mutations in leukodystrophy genes affect integral cellular functions involved in recycling (peroxisomal and lysosomal metabolism), energy production (mitochondrial electron transport), structural integrity (of myelin, cytoskeleton, extracellular matrix, blood brain barrier (BBB), *etc.*), and protein synthesis (messenger ribonucleic acid (mRNA) transcription and translation), among others. Understanding these pathogenic mechanisms (Fig. 2) is critical in designing successful gene therapies.

### Therapeutic modalities for gene delivery

The therapeutic efficacy of gene therapies relies heavily on the efficient delivery of nucleic acid to the nucleus. Viral-mediated gene delivery is predominantly used in clinical trials today for its high efficiency and relative safety. The delivery route also impacts therapeutic efficacy and differs for different leukodystrophies and target cell types. In CNS targeting, the need to traverse the BBB is a fundamental obstacle. In the healthy CNS, astrocytes, pericytes, endothelial cells, and other cell types contributing to BBB structural integrity work together to create a controlled and dynamic microenvironment that maintains BBB integrity and CNS homeostasis [7]. While the integrity of a healthy BBB can impede delivery of gene therapy, pathologic neuroinflammation can disrupt the BBB and alter the pharmacokinetics of viral vector penetration of the CNS [8]. For most CNS disorders (Table 2), common routes of delivery for CNS-targeted gene therapies include *ex vivo* delivery to hematopoietic stem cells (HSCs) [9] and *in vivo* delivery directly to the CNS [10] through intravenous (IV), intracerebral (IC), and/or intrathecal (IT) injections. In the following section, we discuss the different modalities of gene therapy in greater detail.

**Table 2:** Overview of existing gene therapy clinical trials for primary leukodystrophies.

Trial ID	# Patients Enrolled	Intervention/Treatment	Targeted Patient Population	Phase	Start date - completion date
**Metachromatic leukodystrophy**					
NCT01560182	20	IV HSC-directed LV gene therapy (arsa-cel)	Pre-sx or early sx LI or EJ MLD	Phase 1/2	2010–2025
NCT03392987	10	IV HSC-directed LV gene therapy (arsa-cel)	Pre-sx or early sx LI or EJ MLD	Phase 2	2018–2028
NCT04283227	6	IV HSC-directed LV gene therapy (arsa-cel)	Pre-sx or early sx LJ MLD	Phase 3	2022–2031
NCT02559830	50	IV HSC-directed LV gene therapy	Age of symptom onset <16 yrs, non-end stage MLD	Phase 1/2	2015–2025
NCT03725670	10	IC LV gene therapy	All MLD patients with abnormal bMRI	Not reported	2018–2020
NCT01801709	5	IC AAV gene therapy (AAVrh.10cuARSA)	Early-onset MLD ages 6 mo to 5 yrs	Phase 1/2	2014–2029
**X-linked adrenoleukodystrophy**					
NCT01896102	32	IV HSC-directed LV gene therapy (eli-cel)	Active CALD ages ≤17 yrs	Phase 2/3	2013–2021
NCT03852498	35	IV HSC-directed LV gene therapy (eli-cel)	Active CALD ages ≤17 yrs	Phase 3	2019–2024
NCT02698579	67	IV HSC-directed LV gene therapy (eli-cel)	Having received eli-cel in parent study	Long term follow-up	2016–2037
NCT02559830	50	IV HSC-directed LV gene therapy	Age of symptom onset <16 yrs, non-end stage X-ALD	Phase 1/2	2015–2025
NCT03727555	10	IC LV gene therapy	All symptomatic X-ALD patients, NFS≥1	Not reported	2018–2020
NCT05394064	16	IT AAV9 gene therapy (SBT101)	All AMN patients able to ambulate	Phase 1/2	2022–2029
**Globoid cell leukodystrophy**					
NCT04693598	6	IV CNS-directed AAVrh.10 gene therapy (FBX-101)	EI GLD patients, received allogeneic HSCT	Phase 1/2	2021–2024
NCT05739643	12	IV CNS-directed AAVrh.10 gene therapy (FBX-101)	Infantile GLD patients, received allogeneic HSCT	Phase 1/2	2023–2025
NCT06308718	25	IV CNS-directed AAVrh.10 gene therapy (FBX-101)	All patients who received FBX-101	Long term follow-up	2024–2029
NCT04771416	24	ICM AAV.Hu68 gene therapy (PBKR03)	EI GLD patients ages 1–9 mo old	Phase 1/2	2022–2030
**Canavan disease**					
NCT04833907	24	ICV AAV gene therapy (rAAV-Olig001-ASPA)	CD patients ≤60 mo old	Phase 1/2	2021–2024
NCT04998396	18	IV AAV9 gene therapy (BBP-812)	CD patients ≤30 mo old	Phase 1/2	2021–2028
NCT05317780	1	IV and ICV dual-route AAV gene therapy (AAV9-CB6-ASPA)	Single 6-mo-old male CD patient	Single-patient investigational new drug	2022- N/A
**Giant axonal neuropathy**					
NCT02362438	21	IT AAV9 gene therapy (scAAV9/JeT-GAN)	GAN patients ≥3 years old	Phase 1	2015–2030
**GM2 gangliosidoses**					
NCT04669535	11	Intrathalamic and ICM gene therapy (AXO-AAV-GM2)	TSD or SD patients between 6 mo and 12 yrs old	Phase 1	2021–2024
**Alexander disease**					
NCT04849741	73	IT ASO (ION373)	AxD patients between 2 and 65 years old	Phase 1/2/3	2021–2025
**Pelizaeus-Merzbacher disease**					
NCT06150716	24	IT ASO (ION356)	Male PMD patients between 2 and 17 yrs old	Phase 1b	2023–2028

Abbreviations: ASO: antisense oligonucleotides; AxD: Alexander disease; bMRI: brain magnetic resonance imaging; CALD: cerebral adrenoleukodystrophy; CNS: central nervous system; EI: early infantile; EJ: early juvenile; GAN: giant axonal neuropathy; GLD: globoid cell leukodystrophy; HSC: hematopoietic stem cell; IC: intracranial; ICM: intra-cisterna magna; ICV: intracerebroventricular; IT: intrathecal; IV: intravenous; LI: late infantile; LV: lentivirus; MLD: Metachromatic leukodytrophy; mo: months; PMD: Pelizaeus-Merzbacher disease; SD: Sandhoff disease; Sx: symptomatic; TSD: Tay-Sachs disease; X-ALD: X-linked adrenoleukodystophy; yrs: years.

#### *Ex vivo* hematopoietic stem cell-directed gene therapy

**Fig. 3:**
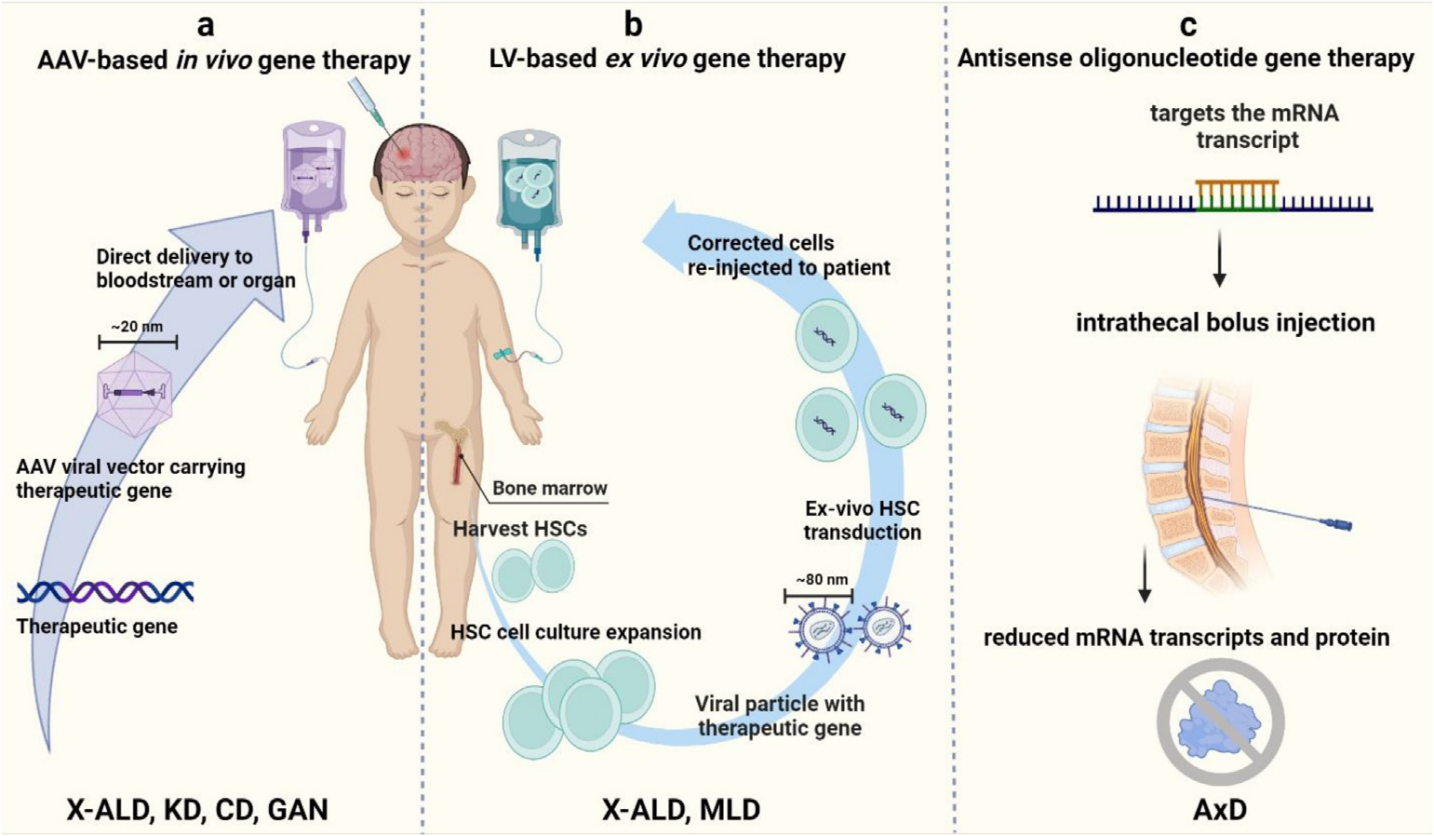
Depiction of the different modalities of gene-based therapies currently in use for leukodystrophy clinical trials, including *ex vivo* LV-mediated gene therapies (left panel), *in vivo* AAV-mediated gene therapies (middle panel), and antisense oligonucleotide therapies (right panel). Abbreviations: X-ALD: X-linked adrenoleukodystrophy, MLD: Metachromatic leukodystrophy, GLD: Globoid cell leukodystrophy, CD: Canavan disease, GAN: Giant axonal neuropathy; TSD: Tay-Sachs disease; SD: Sandhoff disease; PMD: Pelizaeus-Merzbacher disease.

*Ex vivo* HSC-directed gene therapy provides gene replacement to peripherally mobilized HSCs, after which the corrected cells are returned to the patient through autologous hematopoietic stem cell transplantation (HSCT) (Fig. 3, left panel). Recombinant lentiviral (LV) vectors have undergone multiple generations of engineering to enhance safety and efficiency of HSC-directed gene delivery [11] and they are the viral vector of choice for *ex vivo* HSC-directed gene therapy.

Much of the data supporting HSC-directed *ex vivo* gene therapy derive from allogeneic HSCT in the forms of bone marrow transplantation and umbilical cord blood transplantation (UCBT). Over the last few decades, allogeneic HSCT has become the standard of care for many leukodystrophies [12], [13], [14], [15], [16], [17], [18], [19], [20], [22]. It effectively provides gene replacement in the CNS by engrafting donor-derived monocytes as healthy microglia-like cells in the CNS to correct pathologic neuroinflammation mediated by diseased microglia [23], [21]. This therapeutically targets CNS disease through the more easily accessible hematopoietic system. For leukodystrophies successfully treated with allogeneic HSCT, there is strong support for *ex vivo* HSC-directed gene therapy approaches even when animal disease models do not exist.

Unlike allogeneic HSCT, *ex vivo* LV-mediated gene therapy drives supraphysiologic gene expression in transduced autologous HSCs and their progeny. For some leukodystrophies, supraphysiologic gene expression can enhance the therapeutic efficacy of HSCT [24], but for others, supraphysiologic gene expression may have unforeseen cytotoxic effects that preclude its clinical use [25,26]. For leukodystrophies that fall into the former category, *ex vivo* HSC-directed gene therapy can replace allogeneic HSCT and eliminate two critical disadvantages inherent to allogeneic HSCT: the search for a compatible donor and the complications associated with chronic immunosuppression.

Although LV-mediated gene delivery is a powerful tool, there are important limitations and drawbacks to consider. The most important limitation is genomic integration of LV vectors [27]. While stable integration allows for long-term gene expression, integration-related insertional mutagenesis at cancer-associated loci can have significant oncogenic potential [28], especially at high transduction efficiencies that result in high vector copy number per transduced cell.

Another major limitation is the nine kilobase (kb) packaging size of LV vectors. Although this enables packaging of larger genes, it also limits diffusivity in the extracellular matrix and reduces CNS biodistribution with *in vivo* delivery. This is especially limiting in larger animal models and humans [29]. While some preclinical studies show increased CNS distribution with targeted LV envelope engineering [30], these vectors have not yet reached clinical trials. Thus, LVs are predominantly used for *ex vivo* delivery, enabling a cell-based gene therapy product for the leukodystrophies.

#### *In vivo* central nervous system-directed gene therapy

CNS-directed *in vivo* gene therapy targets primary CNS disorders by directly transducing neurons and other CNS cell types (Fig. 3, middle panel). The overwhelming majority of CNS-directed *in vivo* gene delivery is mediated by recombinant adeno-associated virus (AAV) vectors. AAV is a single-stranded DNA parvovirus with a diverse array of naturally occurring and engineered serotypes. By pairing the AAV2 genome with capsids from different serotypes, it is possible to greatly enhance CNS transduction by targeting neurons (AAV9 [[31], [32], [33], [34]], AAVrh.10 [35,36]) and oligodendrocytes (AAV-Olig001 [37], AAVhu68 [38]). Other important CNS-specific AAV capsid features include: 1) BBB penetrance, which allows less invasive IV delivery of CNS-targeted gene therapies [39,40]; 2) retrograde and anterograde axonal transport [41,42], which increases CNS transduction and targets leuko-axonopathies; and 3) non-human primate vector origin, which overcomes pre-existing human AAV immunity. Recombinant AAV serotypes specific to each clinical trial will be discussed in greater detail in the ensuing sections.

A major limitation of existing AAV capsids is their inability to transduce microglia efficiently. Although recent publications have made progress in microglial tropism [43], these technologies have not yet been approved for clinical use. Currently, HSC-directed gene therapy remains the most efficient way to target microgliopathies by allowing corrected monocyte-derived microglia to populate the CNS.

The promoter is another important factor to consider when designing AAV vectors for gene therapy. Some promoters induce supraphysiologic and ubiquitous transgene expression [44,45]. Others have lower expression levels but may have other desired features, such as smaller size, which facilitates accommodation of larger transgenes without exceeding the 4.7 ​kb AAV packaging limit [46].

Compared to LV vectors, AAV vectors are classically considered to be much safer gene delivery vehicles. Instead of integrating into the human genome, AAV mediates long-term gene expression mainly by persisting in episomal form [47], which at least in theory reduces the risk for genotoxicity. However, there are three primary disadvantages associated with AAV-mediated gene therapy: 1) the limited 4.7 ​kb AAV packaging capacity, 2) pre-existing anti-AAV antibody titers that have already excluded up to 20% of patients from receiving life-saving therapy [48], and 3) immunotoxicity associated with high dose AAV administration, which necessitates treatment with immunosuppressive medications [49].

#### Antisense oligonucleotides

Beyond viral vectors, antisense oligonucleotide (ASO) therapy is another form of gene therapy that is predominantly used to modify gene expression at the ribonucleic acid (RNA) level (Fig. 3, right panel). ASOs are synthetic, short, and single-stranded oligodeoxyribonucleotides or oligoribonucleotides that bind to transcribed mRNA via complementary “antisense” base pairing [50,51]. In doing so, ASOs alter pre-mRNA splicing, mRNA stability, mRNA translation, and RNA-protein interactions [[12], [13], [14]]. There are currently two ASOs in clinical trials for Alexander disease and Pelizaeus-Merzbacher disease, leukodystrophies with toxic gain-of-function mutations. ASOs targeting these disorders downregulate expression of the mutant proteins by suppressing mRNA expression.

## Gene therapy clinical trials for leukodystrophies

Within the leukodystrophies, there are active gene therapy clinical trials for metachromatic leukodystrophy, adrenoleukodystrophy, globoid cell leukodystrophy, Canavan disease, giant axonal neuropathy, GM2 gangliosidoses, Alexander disease, and Pelizaeus-Merzbacher disease (Table 2). Here, we provide a brief overview of the pathophysiology and clinical features for each leukodystrophy. Then we present the seminal preclinical gene therapy studies and the existing clinical trials derived from these studies. Because many gene therapies share a similar profile of serious adverse events, we postpone discussion of adverse events to the next section.

### Metachromatic leukodystrophy

#### Pathophysiology and clinical presentation

Metachromatic leukodystrophy (MLD) is an autosomal recessive lysosomal storage disorder that presents in patients who have a deficiency in arylsulfatase A (ARSA) activity. ARSA hydrolyzes sulfatides, and accumulation of sulfatides in MLD destabilizes myelin and impairs oligodendrocytes and Schwann cells, thereby causing a demyelinating leukodystrophy of the CNS and PNS [52,53].

MLD is clinically classified according to age at disease onset: late infantile onset (≤2.5 years), juvenile onset (2.5–16 years), and adult onset (≥16 years) variants [53]. Patients with higher residual enzyme activity are more likely to have milder disease while patients with no or minimal ARSA activity have more severe disease. Late-infantile MLD patients experience rapidly progressive clinical deterioration involving developmental regression, spastic quadriparesis, loss of fine motor control and communication, blindness, deafness, seizures, and death around five to eight years. Juvenile onset MLD initially manifests with psychiatric, cognitive, learning, and attentional problems, followed by spasticity and weakness related to upper motor neuron dysfunction. Patients often live for years in a vegetative state with supportive care. Adult-onset MLD initially presents with psychosis, depression, mood swings, and other neuropsychiatric abnormalities. Over many years, patients experience periods of relative disease stability followed by episodes of exacerbation involving loss of motor skills, bowel and bladder control, speech, and cognition. Death typically occurs 20–30 years after initial diagnosis.

#### Lentivirus-mediated HSC-directed gene therapy

##### Preclinical studies

The therapeutic success of allogeneic HSCT [24,54,55] prompted the first preclinical *ex vivo* HSC-directed LV-mediated gene therapy study [56] in an MLD mouse model that recapitulates the biochemical features of human MLD with some parallels in neuropathologic findings and a milder disease phenotype [57]. By transplanting syngeneic transduced donor MLD mouse HSCs into lethally irradiated MLD recipient mice, this seminal study provided proof-of-concept for the therapeutic viability of *ex vivo* HSC-directed gene therapy by showing: 1) sustained bone marrow engraftment of donor HSCs with supraphysiologic ARSA activity; 2) multilineage hematopoietic reconstitution with transgene-expressing progeny cells; 3) CNS and PNS engraftment of donor-derived cells; and 4) amelioration of the biochemical, neuropathologic, and clinicobehavioral features of murine MLD [56]. Interestingly, the degree of clinical efficacy for *ex vivo* LV-*ARSA* gene therapy appeared to be proportional to the degree of LV-mediated *ARSA* overexpression. A follow-up study showed that ARSA produced by donor monocyte-derived microglia-like cells could correct neighboring ARSA-deficient neurons and glia [58] through a physiologic lysosomal enzyme trafficking process known as cross-correction [59].

##### Clinical trials

Following two open-label, non-randomized phase 1/2 human clinical trials with expanded access for atidarsagene autotemcel (arsa-cel) (NCT01560182, NCT03392987), this approach received FDA approval in 2024. Arsa-cel is an *ex vivo* HSC-directed LV-mediated gene therapy for pre-symptomatic late-infantile, presymptomatic juvenile, and early symptomatic juvenile MLD patients. Interim data analysis at three years of median follow-up time showed biochemical disease correction with persistent supraphysiologic ARSA activity in peripheral blood mononuclear cells (PBMCs). Persistent physiologic ARSA activity in the cerebrospinal fluid (CSF) was indirect evidence for CNS engraftment of donor cells. Treated patients had significantly fewer demyelinating lesions on brain MRI compared to the natural history cohort. This was associated with improvements in gross motor function, increased event-free survival, and normalization or near-normalization of cognitive performance and verbal skills [[60], [61], [62]]. At a median follow-up of 6.76 years, 25/26 treated patients continued to have preserved ambulation [63]. In contrast, 50% of untreated late infantile MLD patients lose their ability to walk a year after symptom onset [64]. Arsa-cel is now being evaluated in a clinical trial for late-juvenile MLD patients (NCT04283227).

Despite these exciting data, not all MLD patients had positive outcomes in the clinical trials. Two early juvenile MLD patients with pre-existing mild cognitive or gait difficulties died shortly after gene therapy infusion due to rapid disease progression. This lends further support to previously published data in allogeneic HSCT suggesting that the presence of pre-treatment neurologic symptoms is a predictor of poor treatment outcomes [13,14,65]. The availability of a disease-modifying therapy will help implement newborn screening, which will in turn facilitate early diagnosis and gene therapy administration in the presymptomatic phase, thereby giving patients the best chance for maximal therapeutic effect.

The short follow-up period for arsa-cel limits its direct comparison to allogeneic HSCT outcomes. Published data suggest a trend toward improved survival, motor function, and cognitive function for patients receiving arsa-cel [12,13,24]. Autopsy studies for MLD patients who received allogeneic HSCT confirmed the presence of CNS donor monocyte-derived microglia-like cells and found evidence of remyelination despite a surprising lack of CNS cross correction [21,66]. Although there are no published autopsy data for MLD patients who received arsa-cel, the pre-clinical murine studies [56,58] suggest that CNS cross-correction is exclusively associated with LV-mediated supraphysiologic *ARSA* expression. Thus, transduced donor cell-mediated cross-correction of neuronal and glial cells may be responsible for the observed improved clinical outcomes of arsa-cel. Additional studies will be needed to test this hypothesis.

A similar *ex vivo* HSC-directed LV-mediated gene therapy is being conducted in China for all MLD patients with disease onset ≤16 years (NCT02559830). Unlike the clinical trials presented above, this clinical trial targets symptomatic MLD patients, and presymptomatic disease is an exclusion criterion. No published data are available for this trial.

#### LV-mediated CNS-directed gene therapy

LV-mediated gene therapies are limited by lack of widespread CNS biodistribution due to large viral particle size and resulting low diffusivity within the extracellular space. This limitation is less apparent in mice due to their small brain size. Intracerebral LV-*ARSA* prevented local hippocampal neuropathology in MLD mice, which corresponded to a small but significant reduction in learning impairment [67]. Since as little as 5% residual ARSA activity can prevent symptomatic MLD [68,69], there is a clinical trial in China studying CNS-directed LV-*ARSA* gene therapy for MLD patients (NCT03725670). However, no trial-related data have been published to date.

#### AAV-mediated CNS-directed gene therapy

One limitation of HSC-directed gene therapy is the inevitable delay between gene therapy infusion and CNS engraftment. This explains the observed 12- to 24-month delay between allogeneic HSCT and any measurable clinical therapeutic benefit [55]. This also explains why more advanced MLD patients derive no benefit from allogeneic HSCT. Direct IC gene therapy could facilitate rapid supraphysiologic transgene expression in the CNS and potentially arrest CNS demyelination without delay.

##### Preclinical studies

Following up on promising preclinical studies using the older generation AAV5 serotype [70,71], Piguet et al. directed a single AAVrh.10-*ARSA* injection targeting the right striatum in MLD mice, which resulted in widespread supraphysiologic ARSA activity in the ipsilateral hemisphere and low but detectable *ARSA* expression in the contralateral hemisphere [35]. While behavioral data for MLD mice treated with AAVrh.10-*ARSA* are lacking, treatment with AAV5-*ARSA* at the presymptomatic stage improved motor function [71]. Compared to AAV5, AAVrh.10 showed wider supratentorial transduction, increased transgene expression, and cross correction of neighboring oligodendrocytes [35]. This justified the translation of IC AAVrh.10-*ARSA* gene therapy to clinical trial.

##### Clinical studies

Despite the promising preclinical efficacy data in mice and the favorable biodistribution and biosafety data of the AAVrh.10-*ARSA* vector in nonhuman primates (NHPs) [72], an open-label, single-arm, monocentric phase 1/2 trial to evaluate the safety and efficacy of IC AAVrh.10*-ARSA* administration (NCT01801709) showed no clinical benefit for presymptomatic or early-symptomatic MLD patients [73].

The failure of this clinical trial is unexpected and unexplained. However, there are a few possible contributors to consider. Human brains have a volume of around 1400 ​cm^3^ [74], whereas adult mouse brains average around 2 ​cm^3^ [75]. The 700-fold difference in brain sizes may have resulted in inadequate viral transduction, CNS biodistribution, and cross correction of brain cells critical for disease modification. Follow up toxicity studies in NHPs identified parenchymal neuroinflammation with microglial activation and CNS infiltration of lymphocytes and monocyte-derived microglia-like cells at a dose of 1.3 ​× ​10^11^ viral genomes (vg) per injection site for both AAVrh.10-*ARSA* and empty (non-transgene-encoding) AAVrh.10 vectors [76]. The higher dose used in the clinical trial (3.3 ​× ​10^11^ vg/site) exceeds the toxic dose and the lower dose (8.3 ​× ​10^10^ vg/site) is higher than the identified maximal safe dose (2.4 ​× ​10^9^ vg/site). In MLD pathophysiology, abnormal microglial activation precedes myelin destruction [77]. AAV-related neuroinflammation may further decompensate an intrinsically vulnerable microglial population and precipitate and/or accelerate MLD disease progression. This paradoxical AAV-mediated acceleration of pathologic microglial activation with associated lymphocytic infiltration has been previously reported in a different leukodystrophy mouse model with progressive disease-associated neuroinflammation [78].

### X-linked adrenoleukodystrophy

#### Pathophysiology and clinical presentation

X-linked adrenoleukodystrophy (X-ALD) is a demyelinating peroxisomal disorder that classically presents in boys and men with hemizygous mutations in the ATP-binding cassette subfamily D member 1 (*ABCD1*) gene. *ABCD1* encodes adrenoleukodystrophy protein (ALDP), a transporter for very long-chain fatty acids (VLCFAs) to the peroxisome where they undergo β-oxidation [79]. There are two neurologic phenotypes associated with X-ALD, cerebral ALD (CALD) and adrenomyeloneuropathy (AMN). CALD is a severe demyelinating phenotype in the brain that develops in 35% of X-ALD boys ≤10 years old. Boys with CALD present with visual and auditory processing difficulties, loss of communication, personality changes, spastic quadriparesis, tube feeding dependence, urinary and fecal incontinence, and seizures. Death typically occurs two to five years after symptom onset. AMN is the most common X-ALD phenotype characterized by an insidious progression of spinal cord and peripheral nerve demyelination. Patients with AMN present in their 20s–30s with progressive spastic paraparesis, sensory loss, neurogenic bladder, and sexual dysfunction. Around 45% of AMN patients eventually develop CALD [80,81].

How *ABCD1* deficiency causes a demyelinating phenotype in CALD is poorly understood, although it is thought to involve a pathologic positive feedback loop driven by VLCFA accumulation, which damages tissue, increases oxidative stress, and promotes neuroinflammation [79]. Neuropathologic analysis of brain tissue from CALD patients shows evidence of a pathologic process initiated by microglial activation and apoptosis and ending with a confluent necrotic white matter lesion with no remaining oligodendrocytes, myelin, or axons [82].

#### Lentivirus-mediated HSC-directed gene therapy

##### Preclinical studies

There is no animal model of CALD, precluding preclinical studies of therapeutic efficacy. Instead, preclinical studies used human LV-*ABCD1* transduced HSCs to show successful human HSC engraftment in the bone marrow [83] and CNS [84] of nonobese diabetic/severe combined immunodeficient recipient mice. These studies provided proof-of-concept for HSC-directed LV-*ABCD1* gene therapy, directly enabling clinical trials. It is important to recognize that the lack of an animal CALD model did not hinder the clinical translation of this therapeutic approach to patients in need. Instead, clinical translation was supported by the success of allogeneic HSCT in the same disease context.

##### Clinical studies

Building on the therapeutic success of allogeneic HSCT [15,16,85,86], four CALD boys with no HLA-compatible HSC donors underwent the first *ex vivo* LV-*ABCD1* gene therapy. These patients initially experienced clinical stabilization and discontinuation of active demyelination starting 14–16 months after gene therapy infusion, but three of the four patients eventually experienced severe neurologic decompensation in the setting of disease progression [87,88]. The eventual disease progression was attributed to low viral genome copies and low ALDP expression. Since then, there have been larger phase 2/3 and long-term follow-up clinical trials in the United States (NCT01896102, NCT03852498, NCT02698579), as well as a small phase 1 clinical trial in China (NCT02559830) to assess clinical efficacy and safety of LV-*ABCD1* gene therapy. We review data derived from the US clinical trials as there are no published data available from the Chinese trial.

The US clinical trials for elivaldogene autotemcel (eli-cel) enrolled boys ≤17 years who presented with early symptomatic CALD, defined as radiologic Loes score 0.5 to nine [89] and clinical neurologic functional scale (NFS) score ≤1 [90]. Presymptomatic X-ALD boys were excluded as it is impossible to predict which patients will develop the CALD phenotype. Fifteen of the 17 patients included in the interim analysis were alive without major functional disabilities or clinical symptoms with a median follow-up time of 29.4 months. Twelve treated patients had stable MRI findings without progression and two had increasing Loes scores with relative preservation of the NFS score. The results of this initial interim analysis [91] were largely confirmed in a subsequent interim analysis with 32 treated CALD patients [92]. Despite the strong clinical and imaging evidence for disease stabilization and biochemical evidence for *ABCD1* transgene expression at a level twice as high as the initial French clinical trial [88], there was no decrease in plasma VLCFA levels. This is consistent with the known lack of correlation between VLCFA levels and disease severity [93]. Further, it suggests that <25% median restoration of ALDP expression in monocytes and macrophages is not enough to reduce plasma VLCFAs, although it is enough to rescue CNS disease. Based on these data, treatment of a focal CNS lesion is not contingent on restoration of VLCFA levels in plasma.

Of the two patients who died in the study, one experienced rapid disease progression immediately after eli-cel administration, possibly due to advanced disease pre-infusion given the expected 12-month period of disease progression between eli-cel administration and disease stabilization. The other patient was withdrawn from the study due to disease progression on imaging, and he unfortunately succumbed to complications related to a second allogeneic HSCT that was deemed unrelated to eli-cel administration. We discuss insertional mutagenesis in the eli-cel gene therapy trial in detail in a subsequent section.

##### LV-mediated intracerebral-directed gene therapy

There is an ongoing Chinese clinical trial studying CNS-directed LV-mediated gene therapy for CALD patients (NCT03727555), although no trial-related data have been published to date. The same limitations for CNS-directed LV administration we previously discussed for MLD apply to CALD.

##### AAV-mediated intrathecal-directed gene therapy

AMN is the most common X-ALD phenotype and the myeloneuropathic component affects both men and women [94]. There is a large clinical need and no available treatments targeting AMN. Single dose IT delivery of AAV9-*ABCD1* in the mouse model of AMN [31] results in widespread transduction of neurons, astrocytes, vascular endothelial cells, and some microglia in the spinal cord. Importantly, the dorsal root ganglion, a major contributor to the progression of sensory abnormalities afflicting patients with AMN [95], is transduced, although there is no reduction of VLCFA accumulation [31]. A subsequent study of AAV9-*ABCD1* targeting the intracerebroventricular (ICV) CSF space showed correction of neuropathologic abnormalities and functional motor improvement in AMN mice [96]. Importantly, IT administration of AAV9 vectors transduced the spinal cord and several brain regions with high efficiency in NHPs [97], demonstrating clinical translatability.

##### Clinical studies

A phase 1/2 randomized, blinded, dose-escalation study of IT delivery of SBT101 (AAV9-*ABCD1*) is enrolling independently ambulating adult men between 18 and 65 years old who have clinical evidence of AMN (NCT05394064). Dose selection was informed by murine and NHP biodistribution studies that were translated to human patients based on relative volumes of CSF [98]. Intrathecal administration of AAV-mediated gene therapy could lower the risk for adverse events by requiring much lower doses of AAV (1.0 ​× ​10^14^ to 3.0 ​× ​10^14^ vg/person) compared to systemic IV administration (as high as 3.0 ​× ​10^14^ vg/kg [99]). There are no interim data for SBT101 published to date.

### Globoid cell leukodystrophy

#### Pathophysiology and clinical presentation

Globoid cell leukodystrophy (GLD) is a demyelinating leukodystrophy that presents in patients with autosomal recessive mutations in the *GALC* gene. *GALC* encodes galactosylceramidase (GALC), the lysosomal enzyme that hydrolyzes the metabolite, galactosylsphingosine, also known as psychosine [100]. Psychosine forms as a cytotoxic byproduct of myelin synthesis and accumulates primarily in the lipid rafts of oligodendrocytes, where it alters oligodendrocyte membrane architecture [101] and induces apoptosis of oligodendrocytes and Schwann cells [102]. The subsequent breakdown of myelin triggers a neuroinflammatory response with microglial and astrocytic activation as well as CNS infiltration of peripheral monocytes. The accumulation of phagocytosed myelin debris in microglia leads to the formation of the pathognomonic giant nucleated “globoid cells.” Cytotoxic psychosine accumulation is directly responsible for the clinical manifestations of GLD, independent of GALC deficiency [103]. In recent years, various important subcellular processes, including autophagy [104], extracellular vesicular trafficking [105], and α-synuclein pathology [106,107] have been implicated in GLD pathophysiology.

GLD is clinically classified based on age of onset: early infantile (0–13 months), late infantile (13–36 months), juvenile (3–16 years), and adult (>16 years). Early infantile GLD presents with irritability, feeding difficulties, optic atrophy, severe spasticity, posturing, and seizures with rapid culmination in death. Late infantile patients develop abnormal gait and visual difficulties, followed by spastic quadriparesis, seizures, temperature instability, and intermittent apneas. On average, patients succumb six years after presentation. Juvenile GLD similarly affects multiple domains, but disease progression occurs more slowly. Adult-onset GLD presents with motor and sensory polyneuropathies, mood alterations, cognitive slowing, and neuropsychiatric manifestations in addition to spasticity, upper motor neuron pattern weakness, and scoliosis over time. The clinical course and prognosis for adults are highly variable [108]. Psychosine accumulation is toxic to peripheral nerves, and a demyelinating peripheral neuropathy is a common and early clinical finding in GLD patients of all ages [109]. Allogeneic HSCT is currently the standard of care but must be completed in the presymptomatic or early symptomatic stages of disease to be effective [19].

#### AAV-mediated intravenous gene therapy

##### Preclinical studies

Recombinant AAV capsids that cross the BBB like AAVrh.10 [110] enable IV delivery of CNS-targeted gene therapies. Although IV delivery is generally not as efficient at transducing the CNS as direct IC delivery, IV delivery does not require general anesthesia and is a much less invasive procedure. GLD mice treated with IV AAVrh.10-GALC had a modest lifespan extension with supraphysiologic levels of GALC activity in the CNS and PNS [111]. The limited therapeutic efficacy of gene therapy, previously reported for other multiple older generation AAV serotypes [[112], [113], [114]], is due at least in part, to persistent pathologic neuroinflammation in the CNS of GLD mice [78]. The presence of AAV exacerbates GLD-associated microglial activation and induces an AAV-targeted lymphocytic infiltration that significantly limits the therapeutic potential of AAV-mediated monotherapy. Interestingly, HSCT drastically reduces both disease-associated and AAV-related neuroinflammation [78,115], and together with gene therapy, produces a synergistic increase in GLD murine lifespan [114,116] that is significantly more than that achieved with AAV monotherapy even at a tenfold higher dose [117]. This synergy has been confirmed in a preclinical study of IV AAVrh.10-GALC and HSCT combination therapy for canine GLD [118].

##### Clinical studies

There are two phase 1/2 non-blinded and non-randomized dose escalation clinical trials for IV delivery of FBX101, an AAVrh.10-mediated gene therapy for *GALC* replacement, in GLD patients who have already had allogeneic HSCT in the form of UCBT. The initial study (NCT04693598) is enrolling early infantile GLD patients only, whereas the second study (NCT05739643) expands patient eligibility to include early and late infantile GLD patients. Interim analysis for the first two treated patients reported increased plasma and CSF GALC activity, improvements in gross motor function, and normalized white matter in treated GLD patients compared to healthy controls [119]. A follow up trial (NCT06308718) is underway to assess the long-term efficacy of FBX-101 over three years.

#### AAV-mediated intra-cisterna magna gene therapy

##### Preclinical data

The cisterna magna is the CSF-filled space dorsal to the medulla and caudal to the cerebellum. Single injection delivery of AAVhu68, an engineered AAV9-derived capsid, to the cisterna magna in NHPs increased CNS transduction of the brain and spinal cord compared to lumbar IT delivery [120]. Treatment of GLD dogs with intra-cisterna magna (ICM)-delivery of AAVhu68-GALC resulted in supraphysiologic GALC activity in the cerebellum and at least physiologic levels in other CNS areas. Treated GLD dogs had decreased neuroinflammation, minimal psychosine accumulation, preserved myelination, restoration of brainstem auditory evoked responses, and normalized nerve conduction velocities (NCVs) [38]. Abnormal NCVs are a pathologic manifestation of human GLD that remains uncorrected with allogeneic HSCT, resulting in progressive peripheral neuropathy with significant functional impairment [20,121]. Correction of this deficit would significantly improve quality of life for GLD patients.

##### Clinical studies

A phase 1/2 clinical trial for ICM delivery of AAVhu68-GALC gene therapy (NCT04771416) was initiated in February 2022. However, this clinical trial was halted by the sponsor in early 2023 due to changes in the company strategy. There are no published data associated with this clinical trial.

### Canavan disease

#### Pathophysiology and clinical presentation

Canavan disease (CD) is an autosomal recessive leukodystrophy that presents in patients with biallelic mutations in the *ASPA* gene. *ASPA* encodes aspartylacylase (ASPA), which catalyzes the hydrolysis of N-acetylaspartic acid (NAA) to aspartate and acetate. Acetate is an important metabolite for myelin formation [122] and abnormal accumulation of NAA promotes oligodendrocyte cell death [123]. However, how this eventually causes myelin vacuolization and spongy degeneration of the brain remains unknown.

CD presents in the first six months of life with hypotonia, head lag, and macrocephaly. Over time, hypotonia evolves into spasticity and patients develop vision loss, seizures, sleep disturbances, irritability, startle response, tonic spasms, fevers of unknown origin, and feeding difficulties [124]. Without treatment, most patients die before age ten. For a small subset of patients, the phenotype is milder with some degree of intellectual impairment, and the disease progression is slower [125].

#### AAV-mediated intracerebroventricular gene therapy

##### Preclinical studies

Most AAV serotypes with CNS tropism primarily transduce neurons, albeit with varying efficiency. Since CD primarily affects oligodendrocytes, CNS-directed AAV-mediated gene therapy with primary neuronal transduction has limited clinical efficacy [126,127]. To target oligodendrocytes, a new AAV capsid with oligodendrocyte-specific tropism, AAV/Olig1, was engineered through capsid shuffling [128]. Although AAV/Olig1 has limited traversal of the BBB [128], direct ICV administration of AAV/Olig1-*ASPA* resulted in widespread preferential transduction of oligodendrocytes primarily in the subcortical white matter. This translated to improved motor function [129] in an ASPA-deficient murine model of CD [123]. Clinical translation of the AAV/Olig1 vector would be a major advance in treating myelinopathies.

##### Clinical studies

A phase 1/2 clinical trial is currently enrolling CD patients 3 months–60 months of age in an open-label sequential cohort study to assess the safety and efficacy of ICV-directed AAV/Olig1-*ASPA* gene therapy (NCT04833907). There are currently no published interim data analyses for this trial.

#### AAV-mediated intravenous gene therapy

##### Preclinical studies

IV delivery of AAV9-*ASPA* to CD mice restored 50% physiologic ASPA activity in the brain, confirming successful CNS transduction [110]. Fifty percent restoration of CNS ASPA activity is sufficient for therapeutic effect, as there is a dramatic 27-fold increase in median lifespan in IV AAV9-*ASPA* treated CD mice compared to untreated CD mice. Despite the lifespan prolongation, there is incomplete restoration of motor function. This is a common therapeutic limitation for gene therapies with multiple possible contributing explanations, including incomplete CNS transduction and/or biodistribution, inadequate cell-specific transduction, and nonphysiologic gene expression. Technologic improvements in gene delivery in the past 20 years have already made remarkable progress in addressing these limitations for CD [130] and other leukodystrophies, and many newer advancements are in preclinical development.

##### Clinical studies

A phase 1/2 clinical trial for BBP-812 is currently enrolling CD patients under 30 months of age in an open-label sequential cohort study to evaluate the safety and efficacy of IV AAV9-*ASPA* gene therapy (NCT04998396). Interim data for four treated patients showed robust NAA reductions in urine, CSF, and brain, with initial suggestions of clinical stabilization [131]. A follow-up open-label, expanded-access clinical trial (NCT05317780) enrolled one CD patient for dual route IV and ICV administration of AAV9-*ASPA*, with subsequent reduction in CSF NAA accumulation, increase in CNS remyelination, progress in neurodevelopment, and reversal of cortical blindness. Although it is difficult to draw conclusions from an n ​= ​1 study, these data provide hope for future dual route gene therapy delivery modalities.

### Giant axonal neuropathy

#### Pathophysiology and clinical presentation

Giant axonal neuropathy (GAN) is an autosomal recessive leuko-axonopathy that affects patients with mutations in the *GAN* gene. *GAN* encodes the gigaxonin protein [132], which is a subunit of E3 ubiquitin ligase that regulates intermediate filament (IF) turnover [133]. Loss-of-function *GAN* mutations result in pathologic IF aggregation in neuronal axons of the CNS and PNS.

Affected patients classically present in infancy or early childhood with distal-predominant sensorimotor peripheral axonal neuropathy and proximal motor weakness. CNS manifestations include developmental delay, cerebellar and pyramidal signs, cranial nerve abnormalities, and seizures [[134], [135], [136]]. Non-neurologic manifestations include kinky hair and long eyelashes [135], precocious puberty [137], skin abnormalities [138,139], gastrointestinal problems [140,141], diabetes [142], and renal tubular acidosis [138]. Most patients succumb to the disease in the third decade in the setting of respiratory failure [143]. A subset of patients with milder GAN phenotypes do not have CNS manifestations or kinky hair [144].

#### AAV-mediated intrathecal gene therapy

##### Preclinical studies

Several *in vitro* preclinical studies have demonstrated significant reductions in IF aggregation in GAN patient-derived fibroblasts [34,145,146] and induced pluripotent stem cell-derived motor neurons [147] after treatment with gene replacement therapy. These data precipitated preclinical studies in two different GAN mouse models [148,149]. ICM administration of AAV9-*GAN* leads to efficient CNS transduction with supraphysiologic *GAN* expression. This correlated with near complete, 50%, and 66% clearance of IF aggregation in the striatum, cortex, and dorsal root ganglia, respectively. Treated GAN mice showed a modest but significant improvement in motor function [34]. The observed magnitude of behavioral improvement may have been limited by the very modest deficits in untreated GAN mice [148].

Transgenic *GAN* expression was driven, not by the commonly used CAG promoter, but by the short JeT promoter to permit packaging of the entire *GAN* coding sequence into the AAV vector. JeT mediates transgene expression at 25% of CAG-mediated transgene expression levels [46], which is acceptable given low physiologic *GAN* expression under healthy conditions [150]. This is a useful strategy for gene replacement of other similarly large genes with low physiologic expression.

##### Clinical studies

A phase 1 open-label non-randomized IT dose escalation study for AAV9-*GAN* has enrolled 14 patients with a median follow-up time of 68.7 months (range 8.6–90.5 months) [151]. Four doses of AAV9-*GAN* were administered in this dose escalation study. Patients receiving the lowest dose did not experience any significant improvement in sensorimotor neuropathy, but patients receiving the three higher doses showed significant improvements in motor function and/or neuropathy severity scores. There was evidence of increased sensory nerve action potential amplitudes across the median and ulnar nerves, which persisted for 6–24 months, indicating successful reversal of sensory neuropathy. Interestingly, the degree of improvement did not correlate with escalating doses. Possible explanations include: 1) the treatment groups were not large enough to detect significant differences, 2) there is a maximum viral dose above which therapeutic efficacy plateaus or diminishes, and/or 3) clinical efficacy of viral-mediated gene delivery correlates not with gene dosage but with other factors such as area of CNS distribution or primary targeted cell type. Additional studies will need to be done to test these hypotheses.

#### GM2 gangliosidoses

##### Pathophysiology and clinical presentation

GM2 gangliosidoses are a group of leuko-axonopathies characterized by deficient degradation of GM2 gangliosides. Affected patients have mutations in the subunits of N-acetyl-β-hexosaminidase (Hex) isoenzymes or in their cofactor, GM2 activator protein. We focus on isoenzyme deficiencies in this review, as they are the targets for current gene therapy approaches. Biallelic mutations in *HEXA*, which encodes the α subunit, cause Tay-Sachs disease (TSD); biallelic mutations in *HEXB*, which encodes the β subunit, cause Sandhoff disease (SD) [152]. Toxic accumulation of GM2 gangliosides primarily occurs in neurons and triggers neuronal apoptosis [153,154] through calcium dyshomeostasis in the endoplasmic reticulum [155]. The mechanisms by which neuronal cell death drive myelin deficiency and hypomyelination remain unknown but are likely related to non-cell autonomous effects of the oligodendrocyte-axon unit.

Clinically, GM2 gangliosidoses are classified based on age of onset. Patients with the acute infantile variant have the most severe clinical course, presenting at three to six months of age with acute neurologic deterioration characterized by weakness, hypotonia, hyperreflexia, developmental regression, vision impairment, exaggerated startle response, seizures, and death before five years of age. Most patients have a cherry red macula on fundoscopic exam [156,157]. The subacute juvenile variant presents with developmental regression at two to five years old, followed by spasticity, loss of ambulation, loss of speech, seizures, and progressive brain atrophy, resulting in death by mid-adolescence [158]. Patients with the late-onset variant present in their teenage years or young adulthood. In addition to gait dysfunction and dysarthria, these patients develop neuropsychiatric manifestations and peripheral neuropathy [159]. Patients with SD but not TSD also have systemic disease manifestations, including hepatosplenomegaly, cardiomegaly, macroglossia, and skeletal abnormalities [152].

#### AAV-mediated intrathalamic gene therapy

##### Preclinical studies

Because Hex subunits α and β dimerize, simultaneous co-expression of both α and β subunits in a stoichiometrically balanced ratio is necessary for optimal production of recombinant Hex in gene replacement approaches for TSD and SD [[160], [161], [162]]. Experimental gene therapies for TSD and SD are studied using the murine SD model, which has a severe phenotype that more accurately mimics the human disease course than the murine TSD model [163,164]. A single hemispheric striatal injection of AAV-*HEXA*/AAV-*HEXB* significantly extended lifespan from four months to over six months [165] in SD mice. However, these mice developed severe unilateral motor deficits due to insufficient correction of the untreated hemisphere. In contrast, bilateral striatal injections extended the median lifespan to >400 days in SD mice, and dual route striatal and cerebellar injections nearly normalized median lifespan to ∼700 days [166]. These data provided proof-of-concept to advance this therapeutic strategy to clinical trials.

Widespread CNS-directed delivery of AAV-mediated gene therapy to large animals remains a challenge due to larger brain sizes. The thalamus is the relay station of the brain, and thalamic targeting of gene therapy increases CNS biodistribution through anterograde [167] and retrograde [168] axonal transport. In the feline SD model, bilateral thalamic AAV1-mediated gene therapy significantly extended feline lifespan and improved gait [169]. However, treated cats mounted an AAV-directed humoral response against the AAV1 capsid. To evade pre-existing antibodies, the *HEXA* and *HEXB* transgenes were packaged into the neurotropic AAVrh.8 capsid [169,170]. AAVrh.8-mediated gene therapy targeting the bilateral thalami and deep cerebellar nuclei effectively corrected biochemical abnormalities, prevented demyelinating lesions, and improved clinical function in SD cats [169,171,172]. Further, bi-thalamic AAVrh.8-mediated gene therapy in a sheep model of TSD [173,174] was comparably effective [175,176].

##### Clinical studies

There is an ongoing phase 1 AAVrh.8-mediated clinical trial for patients with TSD and SD between six months and 12 years of age (NCT04669535). Patients receive equimolar AAVrh.8-*HEXA* and AAVrh.8-*HEXB* gene therapy (AXO-AAV-GM2) delivered to the bilateral thalami and intrathecally. IT dosing is divided into ICM and thoracolumbar injections. We review published data from the first two treated TSD patients [177]. The first patient (TSD-001) presented with infantile-onset TSD at five to six months and received AXO-AAV-GM2 at 2.5 years old. Due to disease-related destruction of the thalami, this patient did not receive intrathalamic AAV, but instead received IT gene therapy only. This patient had significantly impaired neurologic function with a CHOP-INTEND score of 20 prior to treatment, which remained stable without further deterioration 2.5 years after receiving gene therapy.

The second patient (TSD-002) was presymptomatically diagnosed with TSD and had developed lower extremity weakness and a macular cherry red spot when enrolled at six months of age. This patient was treated at seven months of age with bilateral intrathalamic and divided IT AXO-AAV-GM2. TSD-002 had minimal neurologic deficits prior to treatment with a normal CHOP-INTEND score of 60. She experienced temporary disease stabilization with continued CNS myelination on brain MRI before experiencing disease progression starting six months after treatment. She eventually lost the ability to sit or respond to audiovisual stimuli, and she developed new seizures by 24 months of age.

For most leukodystrophies, treatment earlier in the disease course is generally associated with improved outcomes, but the two TSD patients published in this study do not follow this pattern. Moreover, the significant disease progression in TSD-002, who received gene therapy immediately after symptom onset, occurred despite the additional intrathalamic gene therapy this patient received. The additional dose lowered post-gene therapy GM2 ganglioside accumulation in the CSF. Correction of CSF and serum HexA activity was minimal, with <1% physiologic HexA activity in the serum and CSF for both patients, despite intrathecal AAV administration. Although this is a case series with two patients, the results raise some concerns about the sustained therapeutic efficacy of intrathalamic AAV administration for TSD and SD. Additional data will be needed before any clinically meaningful conclusions can be drawn.

### Alexander disease

#### Pathophysiology and clinical presentation

Alexander disease (AxD) is an autosomal dominant astrocytopathy that presents in patients with monoallelic mutations in the *GFAP* gene. *GFAP* encodes glial fibrillary acidic protein (GFAP), the major IF in astrocytes. Mutant GFAP accumulation in astrocytes induces formation of Rosenthal fibers (RFs) [178,179] and alters GFAP metabolism [180]. There is associated astrocyte activation [181], proteosome dysfunction [182], impaired stress response [183], microglial activation [184], lymphocytic infiltration [181], and cellular apoptosis [185,186]. The exact mechanisms by which these pathologic processes induce cytotoxicity and demyelination have not been fully elucidated.

There are two subtypes of AxD that are differentiated by brain imaging features and age of onset. Type I AxD is a rapidly progressive and early-onset phenotype, characterized by a frontal-predominant demyelinating leukodystrophy on imaging. Patients present with motor and cognitive delays, seizures, megalencephaly, and paroxysmal deterioration. Type II AxD is characterized by atrophic white matter disease of the brainstem, cerebellum, and spinal cord. It is an insidiously progressive phenotype that can present at any age with upper motor neuron signs, autonomic features, and ataxia manifesting as bulbar symptoms, palatal clonus, and extraocular movement abnormalities. Later onset phenotypes progress more slowly and patients with adult-onset AxD can survive for >30 years after diagnosis [187,188].

#### Antisense oligonucleotide intrathecal gene therapy

##### Preclinical studies

ASOs treat gain-of-function *GFAP* mutations by suppressing expression of the mutant protein [189]. ICV administration of *GFAP*-targeting ASOs in two rodent AxD models reduced mutant GFAP accumulation and reversed neuropathology [190,191]. In the AxD rat model, which develops a robust clinical phenotype, presymptomatic ASO administration prevented motor abnormalities, and ASO administration five weeks after symptom onset incompletely reversed motor abnormalities. This is the first leukodystrophy gene therapy showing reversal of disease progression after symptom onset [182].

##### Clinical studies

A randomized, double-blind, parallel-assignment phase 1/2/3 clinical trial for IT zilganersen, a *GFAP*-targeting ASO, is enrolling AxD patients between two and 65 years old (NCT04849741). There is also an open-label sub-study for AxD patients under two years old. Like other CNS-targeted ASO clinical trials, the success of this trial will likely depend on the efficiency of cell-specific *GFAP* knockdown and the biodistribution of zilganersen in the human brain [192]. There are no published interim analyses.

### Pelizaeus-Merzbacher disease

#### Pathophysiology and clinical presentation

Pelizaeus-Merzbacher disease (PMD) is a hypomyelinating leukodystrophy presenting in patients with X-linked mutations in the *PLP1* gene. *PLP1* encodes myelin proteolipid protein (PLP) and DM20 through an alternative *PLP1* splice product. Together, PLP and DM20 comprise 60–80% of the total protein content in myelin [193]. The pathophysiology of *PLP1* mutations is heterogeneous due to the different types of pathogenic mutations. Toxic gain-of-function *PLP1* mutations include gene duplications and mutations that disrupt protein folding. The resulting pathologic protein accumulation in the endoplasmic reticulum (ER) activates the ER stress response [194] and overwhelms the unfolded protein response [195], triggering oligodendrocyte apoptosis [196]. This results in the classic PMD clinical constellation of nystagmus, ataxia, dystonia, spastic paraplegia, and cognitive delay. Death occurs in the teenage years in the severe connatal form, but most patients with classic PMD survive into adulthood [197].

Alternatively, patients with *PLP1* null mutations do not have nystagmus but present with a mild hereditary spastic quadriplegia (HSP). More conserved amino acid substitutions in *PLP1* are associated with pure HSP, while less conserved mutations are associated with HSP with CNS hypomyelination.

#### Antisense oligonucleotide intrathecal gene therapy

##### Preclinical studies

*PLP1* duplications occur in 70% of PMD patients [198], and ASO-mediated reduction of *PLP1* expression to physiologic levels would, in theory, be curative. Further, the mild phenotype associated with *PLP1* null mutations implies a wide therapeutic window for titration of *PLP1* expression. ICV delivery of a *PLP1*-targeted ASO in a faithful mouse model of PMD [[199], [200], [201]] partially corrected biochemical, neuropathologic and behavioral deficits [202]. Of note, the incomplete correction of nerve conduction velocity in ASO-treated mice may parallel the peripheral neuropathy phenotype seen in patients with *PLP1* null mutations. Although treated mice continue to experience some limitations in motor function, the 11-fold increase in lifespan demonstrates the clear efficacy of this therapeutic approach. Near-normalization of exploratory behaviors also suggests a significant improvement in quality of life.

##### Clinical studies

An interventional trial to assess the safety, pharmacokinetics, and pharmacodynamics of IT ION356, an ASO targeting *PLP1*, is currently enrolling male PMD patients between two and 17 years old with a *PLP1* duplication (NCT06150716). There are no interim data published for this clinical trial.

## Adverse events

Gene-based therapies have tremendous potential in changing the lives of leukodystrophy patients. However, clinical efficacy must be assessed in the context of therapy-related adverse events. Serious adverse events (SAEs) specific to gene-based therapies most commonly arise from genotoxicity and/or immuntoxicity. In this section, we discuss the pathophysiology and treatment for these SAEs.

### Genotoxicity

#### Recombinant lentivirus

Recombinant lentiviruses derive from retroviruses that randomly integrate into the host genome. In the early 2000s, the FDA halted HSC-directed gene replacement therapy for severe combined immunodeficiency due to T cell leukemias that developed in six patients with retroviral vector integration near proto-oncogenes [[203], [204], [205]]. Although LVs are much safer than retroviruses, long-term transgene expression is reliant on LV integration so insertional mutagenesis and oncogenesis remain risks.

As of spring 2024, seven cases of myelodysplastic syndrome or acute myelogenous leukemia have been reported among more than 70 boys with childhood CALD treated with eli-cel to date [28,206]. Two patients had LV insertion at the *MECOM* locus, a known susceptibility locus for myeloid malignancies [207,208] and an inhibitor of cell cycle progression and differentiation of HSCs [209]. However, not all patients with *MECOM* locus integrations have developed therapy-related myeloid neoplasms (tMN), suggesting that there are likely additional oncogenic factors at play that have yet to be identified.

Preclinical safety data in animal models failed to identify the *MECOM* locus as a common integration site (CIS) in mice treated with LV gene therapy. Further, there was no increased fitness for clones with identified CISs after xenotransplantation into humanized *Rag2*^*−/−*^*Il2rg*^*−/−*^ (Ragγ) mice [210]. These studies were likely limited by poor reconstitution of the myeloid lineage in Ragγ mice [211], which limits assessment of oncogenic potential in myeloid precursor cells. Mutations in oncogenic genes with a variant allele frequency (VAF) as low as 0.5% can give rise to tMNs [212]. The clonal evolution and expansion of a myeloid subclone at such low VAFs take time and may account for the observed one-to two-year latency in tMN diagnosis after LV gene therapy. The short 12-week interval between HSCT and LV integration analysis in the murine preclinical study was likely insufficient to assess for oncogenic potential. Thus, close long-term monitoring of CALD patients after LV gene therapy is critical.

Interestingly, there have been no cases of tMN in MLD patients who received arsa-cel. There are significant differences in the LV constructs between the two clinical trials, and additional studies are needed to determine how differences in LV constructs, transgenes, HSCT preconditioning regimens, and other factors may impact the oncogenic potential of LV-mediated gene therapy.

#### Recombinant AAV

Recombinant AAV is classically known as a nonintegrating virus that maintains long-term transgene expression by persisting in cells in episomal form [213,214]. However, AAV genome integration does occur, albeit at a much lower rate, in mice [215,216], dogs [217], primates [218], and humans [219,220]. Integration in the murine genome at the *Rian* locus induces hepatocellular carcinoma (HCC) in mice [221,222]. Although the murine *Rian* locus is partially syntenic to the human *Rian* locus, no AAV integrations in the human *Rian* locus have been identified to date. Further, there are no reports of human malignancies associated with AAV integration except one case of HCC in a hemophilia B patient who was then found to have multiple other coexisting risk factors for HCC [223]. Given the relatively short follow-up time so far for most AAV clinical trials however, it is imperative to continue close long-term monitoring of all patients receiving AAV-mediated gene therapy.

### Immunotoxicity

#### Recombinant lentivirus

Immunotoxic adverse events are triggered by immune activation against the recombinant virus or the encoded transgene, both of which can be identified as foreign by the patient’s native immune system. *Ex vivo* delivery of LV to HSCs is a widely used delivery strategy that minimizes immune responses and immunotoxic adverse events. The arsa-cel MLD clinical trials reported six events of low-titer transient antibodies against the *ARSA* transgene product with no impact on pharmacodynamics or clinical outcomes [62]. There have been no published reports of antibodies against the *ABCD1* transgene product in the eli-cel clinical trial [91], although continued monitoring will be very important.

#### Recombinant AAV

Although AAV is classically known as a virus with low immunogenicity, immunotoxicity is a concern for patients receiving high dose AAV-mediated gene therapy. Thrombotic microangiopathy (TMA) is an SAE that develops in the setting of an immunotoxic response to very high doses of AAV >10^14^ vg/kg. Many IV AAV-mediated gene therapies in clinical trials are in this high dose range [[224], [225], [226]].

TMA is an endothelial injury syndrome characterized by profound thrombocytopenia due to hepatic platelet sequestration [227] and acute liver injury of varying degrees of severity. The kidneys, pancreas, skin, and heart can also be affected [228]. The pathophysiology of TMA is not fully understood, but the complement system [229] is a primary mediator of the immunotoxic response. Recombinant AAV activates the classical [230] and alternative [231] complement pathways, which induce liver sinusoidal endothelial injury, formation of platelet microthrombi, and hepatic platelet sequestration [227].

Due to the immunotoxic SAEs associated with high dose AAV administration, all patients who receive AAV routinely receive steroids for immunosuppression to prevent acute liver injury. However, steroids alone do not prevent thrombocytopenia, complement activation, or antibody formation [49]. Eculizumab, a monoclonal antibody against C5, has successfully blocked the immunotoxic response associated with TMA [232]. However, these studies were done for HSCT-associated TMA. Additional studies will need to be done to assess therapeutic efficacy in the context of AAV-associated TMA.

## Future directions

Over the last 20 years, gene therapies for leukodystrophies have advanced beyond the preclinical realm and reached the clinical setting. For many, the availability of these therapies is life changing. The field continues to advance rapidly in the development of newer gene therapy delivery technologies that creatively evade SAEs while maximizing the potential to achieve a functional genetic “cure.” Areas that are being actively researched include but are not limited to: 1) increasing CNS transduction and biodistribution with bioengineered viral capsids [233]; 2) improving CNS cell-specific targeting to microglia [[234], [235], [236]] to treat primary and secondary microglial pathologies shared by many leukodystrophies; 3) liver-detargeted capsids to minimize hepatotoxicity [237,238]; 4) immune system evasion to expand treatment access to patients who have pre-existing antibodies against viral vectors [239,240]; etc. Further, the recent advent of precision genome editing enables targeted editing of the native genomic DNA, which maintains physiologic expression of corrected gene products and avoids cytotoxic adverse events related to nonphysiologic gene dosing [241]. Although each of these advances will need to be extensively evaluated for safety and efficacy prior to clinical translation, continued improvement in clinical outcomes is now the reality for many leukodystrophies that were once considered universally fatal and untreatable. Despite the progress, vulnerability in the leukodystrophy population demands increased attention. Adverse events specific to distinct classes of gene therapy are increasingly recognized, and the impact of disease-specific pathophysiology brings further unknowns. Ultimately, therapeutic benefits and risks will need to be evaluated in a disease-specific context.

## Author contributions

Jasna Metovic and Yedda Li performed the literature review and wrote the first draft of the manuscript. Yi Gong edited and provided comments and revisions. Florian Eichler supervised the review and revised and edited the manuscript.

## Declaration of competing interest

F.S. Eichler receives research support from NINDS (R01NS072446, R01NS082331, U54NS115052), institutional contracts from ASPA Therapeutics, Sanofi, Ionis, bluebird bio and Minoryx Therapeutics, and performs personal consulting for Sanofi, ASPA Therapeutics, UpToDate, Leal, Takeda, Atlas Venture, Acadia Pharmaceuticals and SwanBio Therapeutics.
